# Multicentric analytical comparability study of programmed death-ligand 1 expression on tumor-infiltrating immune cells and tumor cells in urothelial bladder cancer using four clinically developed immunohistochemistry assays

**DOI:** 10.1007/s00428-019-02610-z

**Published:** 2019-07-02

**Authors:** Kristina Schwamborn, Johannes U Ammann, Ruth Knüchel, Arndt Hartmann, Gustavo Baretton, Felix Lasitschka, Peter Schirmacher, Till Braunschweig, Robert Tauber, Franziska Erlmeier, Stefanie Hieke-Schulz, Wilko Weichert

**Affiliations:** 1 Institute of Pathology, Technische Universität München, Trogerstr. 18, 81675 Munich, Germany; 2 Roche Pharma AG, Grenzach-Wyhlen, Germany; 3 Institute of Pathology, Uniklinik RWTH Aachen, Aachen, Germany; 4 Institute of Pathology, Universität Erlangen-Nürnberg, Erlangen, Germany; 5 Institute of Pathology, Universitätsklinikum Carl Gustav Carus Dresden, Dresden, Germany; 6 Institute of Pathology, Universitätsklinikum Heidelberg, Heidelberg, Germany; 7 Present Address: Institut für Pathologie, Dres. med., Kaufmann und Wilke, Industriestr 11c, 67063 Ludwigshafen, Germany; 8 Department of Urology, Technische Universität München, Munich, Germany; 9 Institute of Pathology, Technische Universität München and Member of the German Cancer Consortium (DKTK), Partner site München, Munich, Germany

**Keywords:** Programmed death-ligand 1, PD-L1, Urothelial bladder cancer, Immunohistochemistry, Tumor-infiltrating immune cells

## Abstract

Programmed death-ligand 1 (PD-L1) expression on tumor cells (TC) or tumor-infiltrating immune cells (IC) correlated in several studies with PD-L1/programmed death-1 (PD-1) checkpoint inhibitor efficacy. Since June 2018, a positive PD-L1 status is required for atezolizumab or pembrolizumab treatment of patients with advanced or metastasized urothelial bladder cancer, who are ineligible for cisplatin-containing therapy. We examined technical comparability and inter-reader agreement of four clinically developed PD-L1 assays in locally advanced disease. Archived, formalin-fixed, paraffin-embedded sections from 30 patients (73.3% cystectomies, 26.7% transurethral resections) were stained by PD-L1 immunohistochemistry using VENTANA SP142, VENTANA SP263, DAKO 22C3, and DAKO 28-8 at two sites per manufacturers’ protocols and scored blinded at five sites for PD-L1 expression on IC (% per tumor area) and TC (%). Small, non-significant inter-assay differences were observed for IC. For TC, SP142 showed significantly lower staining percentages. Pairwise comparisons revealed − 0.3 to 1.6% differences in adjusted means between assays for IC, and for TC, − 10.5 to − 7.8% (SP142 versus others) and − 1.9 to 2.7% (other comparisons). Inter-reader and inter-assay agreement was moderate to high for both IC and TC. Allocation to binary cutoffs (1%, 5%, 10%) showed substantial to high Kappa agreement scores (0.440–0.923) for IC and TC between assays for each reader. This first multicenter study, with five independent readers blinded with respect to the assay used, suggests that all four currently clinically relevant assays are analytically similar for evaluation of PD-L1-stained IC and three (SP263, 22C3, and 28-8) for PD-L1-stained TC. Inter-observer agreement for trained readers in scoring of both IC and TC positivity was generally high.

**Electronic supplementary material:**

The online version of this article (10.1007/s00428-019-02610-z) contains supplementary material, which is available to authorized users.

## Introduction

Urothelial bladder cancer (UBC) results in approximately 165,000 deaths worldwide annually [1]. In patients with metastatic UBC, median overall survival is approximately 9–15 months following first-line platinum-based chemotherapy and 7–9 months for patients relapsing after platinum-based treatment [2–4]. Cancer immunotherapy is a treatment modality addressing this high medical need in UBC. Immune checkpoints, such as the programmed death-ligand 1 (PD-L1)/programmed death-1 (PD-1) pathway, block the development of active antitumor immune responses [5], and inhibition of this pathway has demonstrated excellent response and survival rates in locally advanced or metastatic UBC [6–9].

Efforts to identify patients most likely to benefit from anti-PD-L1/PD-1 therapy suggest that expression of PD-L1 on tumor cells (TC) and/or tumor-infiltrating immune cells (IC) may correlate with efficacy of anti-PD-L1 therapy in UBC [7, 8, 10, 11], as may other potential biomarkers such as tumor mutational burden [12].

In the USA, the anti-PD-L1 antibody atezolizumab is approved for the treatment of patients with locally advanced or metastatic UBC, ineligible for cisplatin-containing therapy, whose tumors have PD-L1-stained IC, covering ≥ 5% of the tumor area, or ineligible for any platinum-containing therapy regardless of PD-L1 expression [13]. The anti-PD-1 antibody pembrolizumab is also approved in the USA for the treatment of patients with locally advanced or metastatic UBC ineligible for cisplatin-containing therapy whose tumors express PD-L1 (combined positive score ≥ 10) or ineligible for any platinum-containing chemotherapy regardless of PD-L1 status [14]. Two other PD-L1 inhibitors, durvalumab and avelumab, and the PD-1 inhibitor nivolumab are approved for second-line treatment in the USA. In Europe, atezolizumab is approved for the treatment of patients with locally advanced or metastatic UBC after platinum-containing chemotherapy or cisplatin-ineligible patients whose tumors have PD-L1-stained IC covering > 5% of the tumor area. Pembrolizumab is approved in Europe for the treatment of patients with advanced UBC recurrent after platinum-based therapy or those ineligible for cisplatin-containing regimens whose tumors express PD-L1 with a combined positive score ≥ 10, while the PD-1 inhibitor nivolumab is approved for second-line treatment. PD-L1 testing for cisplatin-ineligible patients for atezolizumab and pembrolizumab was imposed by the European Medicines Agency (EMA) and the US Food and Drug Administration (FDA) in June 2018 after early data from two first-line studies suggested decreased survival with single-agent checkpoint inhibitors compared with platinum-based chemotherapy in patients with low PD-L1 expression levels [15, 16].

In light of this requirement for PD-L1 testing in cisplatin-ineligible patients with locally advanced or metastasized UBC, accurate and reproducible measurement of PD-L1 expression of TC and IC is crucial. However, assays for PD-L1 expression were developed and validated independently in clinical studies of different checkpoint inhibitors. Several different assays for PD-L1 expression are available, all of which involve the scoring of immunohistochemically stained tumor sections by trained pathologists, but differences in antibodies used, cell types assessed, scoring systems, and cutoffs, as well as inter-observer variability, suggest that assay results may not be concordant [17]. This in turn may impact therapy decisions.

To address this problem, in this multicenter study, we investigated the technical comparability of four clinically relevant PD-L1 immunohistochemistry (IHC) assays in terms of concordance of the percentage of PD-L1-stained IC (per tumor area) and TC. Additionally, consistency of scoring for each assay between different trained readers was assessed.

The primary objective was to assess the overall technical comparability of the four assays in terms of percentage of PD-L1-stained IC, adjusted for reader effects. Secondary objectives included inter-reader agreement of PD-L1 IC staining for each assay, inter-assay agreement of PD-L1 staining for each assay, and comparability of PD-L1 TC staining overall, inter-assay and inter-reader.

## Materials and methods

Staining for VENTANA SP142 and VENTANA SP263 (Roche Diagnostics, Mannheim, Germany) was performed at the Technical University of Munich, Germany, according to manufacturer’s protocol, on a VENTANA BenchMark Ultra (Roche Diagnostics). Briefly, slides were deparaffinized and incubated with cell conditioning solution (Cell Conditioning 1 [CC1], Roche Diagnostics) at 100 °C for 40 min for VENTANA SP142 and at 95 °C for 64 min for VENTANA SP263. The incubation period with the primary antibody was 16 min for both antibodies. For VENTANA SP142, incubation with primary antibody was followed by incubation with the OptiView Detection and Amplification Kit (Roche Diagnostics). Staining for DAKO 22C3, DAKO 28-8, and pan-cytokeratin (Agilent Technologies, Waldbronn, Germany) was performed at Uniklinik RWTH Aachen, Germany, according to manufacturer’s protocol. Formalin-fixed, paraffin-embedded tissue sections underwent a 3–1 target retrieval procedure using PT Link (Agilent Technologies) and low pH, followed by peroxidase blocking and incubation of the primary antibody, linker, visualization reagent, and DAB chromogen (in both PD-L1 antibodies following the companies’ recommended instructions, included in both kits, PD-L1 IHC 22C3 pharmDX and PD-L1 IHC 28.8 pharmDX, Agilent Technologies). Pan-cytokeratin staining was carried out in a similar way, using a broad-spectrum monoclonal mouse antibody (clone AE1/AE3) and EnVision FLEX Kit (Agilent Technologies). Staining procedures were carried out using Autostainer Link 48 (Agilent Technologies). After hematoxylin counterstain, dehydration, and coverslipping, slides were analyzed.

To select samples for the study cohort, sections from archived, formalin-fixed, paraffin-embedded tissue from patients with locally advanced UBC (*n* = 150) were chosen randomly and whole slides stained for PD-L1 using the VENTANA SP142 (Roche Diagnostics). Forty (26.7%) of the selected cases were transurethral resections of bladder tumors and 110 (73.3%) were from cystectomies. All cases were reviewed by two board-certified pathologists trained on scoring PD-L1 IC with VENTANA SP142 (Wilko Weichert and Kristina Schwamborn), and 30 cases were selected based on PD-L1 expression on IC in invasive cancer areas (< 1%, 1–5%, or > 5%; 10 cases, with approximately 30% cystectomies and 70% transurethral resections each) to resemble distribution of PD-L1 IC expression in the atezolizumab IMvigor210 study (cohort 2) [8]. Sample characteristics are summarized in Table S1 in the supplementary material. Only classical urothelial carcinomas (and no histological subtypes) were included in this study. Representative examples of staining for IC are shown in Fig. S1 in the supplementary material. All serial sections for this study were cut at the Technical University of Munich, Germany, and distributed for further staining to the RWTH Aachen, Germany. To aid in defining the tumor area, serial sections from each case were also stained with a pan-cytokeratin antibody and by hematoxylin and eosin.

For every selected case, whole slides were stained with each assay as well as pan-cytokeratin/hematoxylin and eosin and were distributed to the five university pathology departments at the Technical University of Munich, RWTH Aachen, Heidelberg, Erlangen, and Dresden for assessment. Observers were blinded for the assay used but not for the case. At each site, a board-certified pathologist who had been trained in scoring PD-L1 IC in UBC using the VENTANA SP142 IHC assay (0.5-day digital classroom training) [18] scored each case/assay combination (30 cases × 4 assays = 120 slides). All readers were trained in the proper interpretation and scoring of IC with the VENTANA SP142 assay using a method previously outlined for non-small-cell lung cancer and UBC [19]. The training session was performed using the novel digital platform, PathoTrainer (Pathomation Inc., Antwerp, Belgium), with 75 cases, including a 40-case proficiency exam that required a minimum passing score of 85% (the average proficiency score was 95%). Training was conducted across the dynamic range of PD-L1 positivity. Additionally, training specifically included consensus on the following criteria: IC were identified by morphology and TC by morphology and also pan-cytokeratin staining if required. TC were counted as positive if they showed a membranous staining (complete or incomplete) of any intensity. PD-L1 on TC was scored as the percentage of stained cells; the intensity of staining was not assessed. IC were defined as granulocytes, lymphocytes, and macrophages within the tumor, in the vicinity of the TC nest or in the stroma between two adjacent TC nests. Staining of granulomas was included if they met the criteria mentioned above. Necrotic areas, granulomas, or lymphoid aggregates adjacent (but not directly attached) to, or distant from, the tumor, intravascular IC, and areas showing cauterization artifacts were excluded. IC were included if they displayed any intensity of membranous (VENTANA SP263, DAKO 22C3, and DAKO 28-8) or granular cytoplasmic staining (VENTANA SP142). PD-L1 on IC was scored as the percentage of invasive tumor area occupied with/covered by IC showing PD-L1 staining at any intensity.

**Fig. 1:**
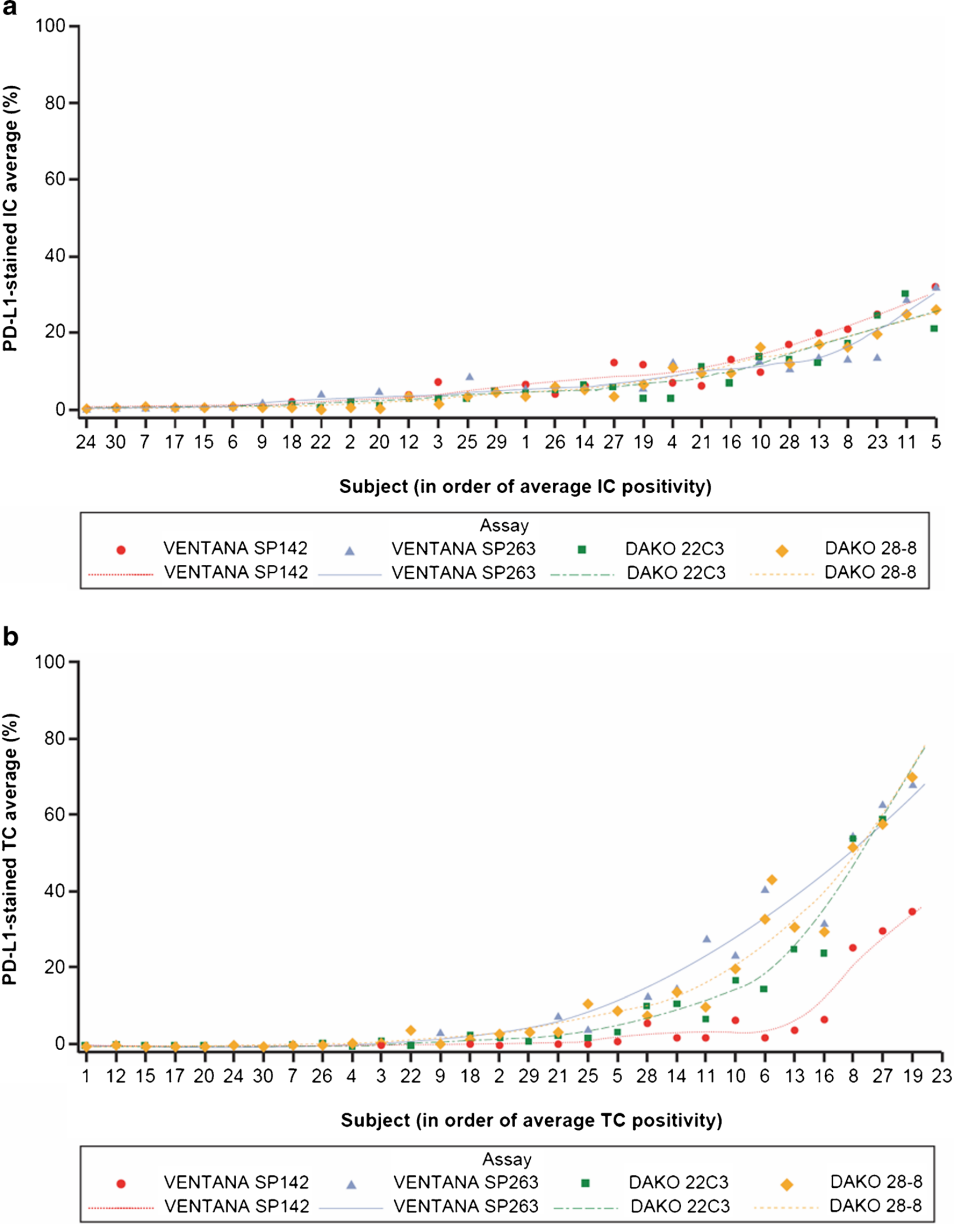
Average percentage of PD-L1-stained IC (**a**) and TC (**b**) using each assay. IC = tumor-infiltrating immune cells; PD-L1 = programmed death-ligand 1; TC = tumor cells

To compare the percentage of PD-L1 staining, an analysis of variance (ANOVA) was conducted using assay, reader and patients as effects. From this model, adjusted mean percentages were obtained for each assay, with 95% confidence intervals for means, and differences estimated and adjusted for multiple comparisons using Tukey’s range test. To allow consideration of the data in the context of the Blueprint study [17], data were visualized after the percentage of PD-L1 staining was averaged over the five readers (Fig. 1).

To investigate inter-reader and inter-assay concordance, intra-class correlations (ICCs) were calculated. The degree of concordance (Fleiss’ Kappa) and averaged percentage disagreement between assays were calculated for retrospectively selected cutoffs for PD-L1 positivity of > 1%, > 5%, and > 10%. The 25% cutoff was not included as too few samples were PD-L1 TC > 25% in the non-enriched screening and study cohort.

## Results

### Percentages of PD-L1-stained IC or TC

Clinicopathological characteristics of the 30 selected cases are shown in Table S1 in the supplementary material. No consistent pattern of higher or lower staining for PD-L1-stained IC was seen for any particular assay, and only small differences between assays were observed (Fig. 1a). The adjusted mean percentage of IC staining for PD-L1 varied from 6.5 to 8.2% depending on the assay used (Table 1). There was broad agreement between readers for each assay, apart from reader 3, who tended to score PD-L1-stained IC slightly higher than the other readers (Fig. S2 in the supplementary material).

**Table 1:** Mean percentages of PD-L1-stained IC and TC across all samples using each assay, adjusted for sample effects

Assay	Adjusted mean PD-L1-stained IC, % (95% CI)	Adjusted mean PD-L1-stained TC, % (95% CI)
VENTANA SP142	8.2 (7.3–9.1)	5.5 (3.7–7.2)
VENTANA SP263	7.1 (6.2–8.0)	15.9 (14.2–17.6)
DAKO 22C3	6.5 (5.6–7.5)	13.2 (11.5–15.0)
DAKO 28-8	6.9 (6.0–7.8)	15.1 (13.4–16.9)

*CI* confidence interval, *IC* tumor-infiltrating immune cells, *PD-L1* programmed death-ligand 1, *TC* tumor cells

In contrast, there was more variation in the percentage of PD-L1-stained TC between assays, with the VENTANA SP142 assay yielding consistently lower percentages than the other three assays (Fig. 1b) and a lower adjusted mean percentage of stained cells (Table 1). There was also more variation between individual readers than for PD-L1-stained IC (Fig. S3 in the supplementary material), with reader 3 again scoring consistently higher than the other readers.

### Pairwise comparison of assays

**Fig. 2:**
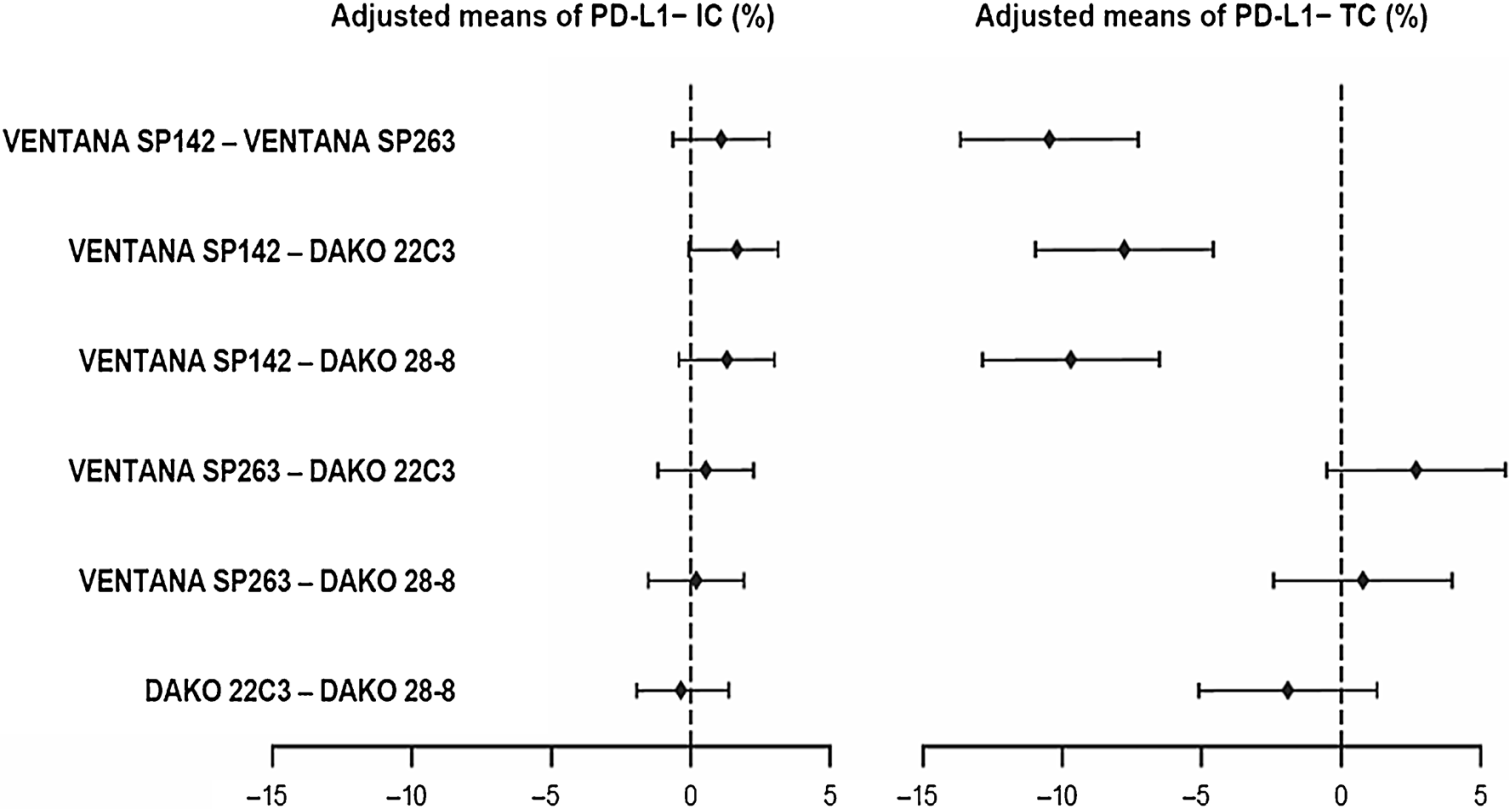
Difference in adjusted means of percentages of PD-L1-stained IC or TC for each assay. IC = tumor-infiltrating immune cells; PD-L1 = programmed death-ligand 1; TC = tumor cells

Pairwise comparison of adjusted means showed small differences between assays in PD-L1-stained IC but larger differences for PD-L1-stained TC, particularly between VENTANA SP142 and other assays (Fig. 2). Mean differences in adjusted means ranged from − 0.3 to 1.6 for IC, and all were non-significant. In regard to TC, staining differences between assays were larger, with wider confidence intervals than for IC (Fig. 2). Differences in adjusted means for TC ranged from − 10.5 to 2.7, with the largest differences being between VENTANA SP142 and the other three assays (range − 10.5 to − 7.8), which were statistically significant. Differences between the three other assays were in the range − 1.9 to 2.7 for TC and were non-significant (Table S2 in the supplementary material).

### Inter-reader and inter-assay agreement

Inter-reader agreement for each assay was moderate to high for IC staining (ICC 0.532–0.729) and for TC staining (ICC 0.609–0.883) (Table 2). For each reader, inter-assay agreement was similarly moderate to high for IC staining (0.681–0.858) and for TC staining (0.778–0.885) (Table 3). This reflects the overall comparability of the assays, and it should be noted that the single outlier (SP142) that was identified in pairwise comparisons cannot be identified by the ICC analysis method.

**Table 2:** ICC values for inter-reader agreement for each assay

Assay	ICC (IC)	ICC (TC)
VENTANA SP142	0.699 (0.561–0.820)	0.609 (0.456–0.759)
VENTANA SP263	0.729 (0.599–0.840)	0.805 (0.701–0.889)
DAKO 22C3	0.532 (0.370–0.700)	0.883 (0.813–0.935)
DAKO 28-8	0.573 (0.413–0.730)	0.845 (0.757–0.913)

*IC* tumor-infiltrating immune cells, *ICC* intra-class correlation, *R* reader, *TC* tumor cells

**Table 3:** ICC values for inter-assay agreement for each reader

Reader	ICC (IC)	ICC (TC)
R1	0.681 (0.528–0.812)	0.850 (0.759–0.917)
R2	0.850 (0.758–0.917)	0.778 (0.655–0.874)
R3	0.858 (0.770–0.992)	0.885 (0.811–0.937)
R4	0.836 (0.737–0.909)	0.784 (0.664–0.878)
R5	0.839 (0.741–0.910)	0.812 (0.703–0.895)

*IC* tumor-infiltrating immune cells, *ICC* intra-class correlation, *R* reader, *TC* tumor cells

### Allocation to binary cutoffs for IC or TC

**Fig. 3:**
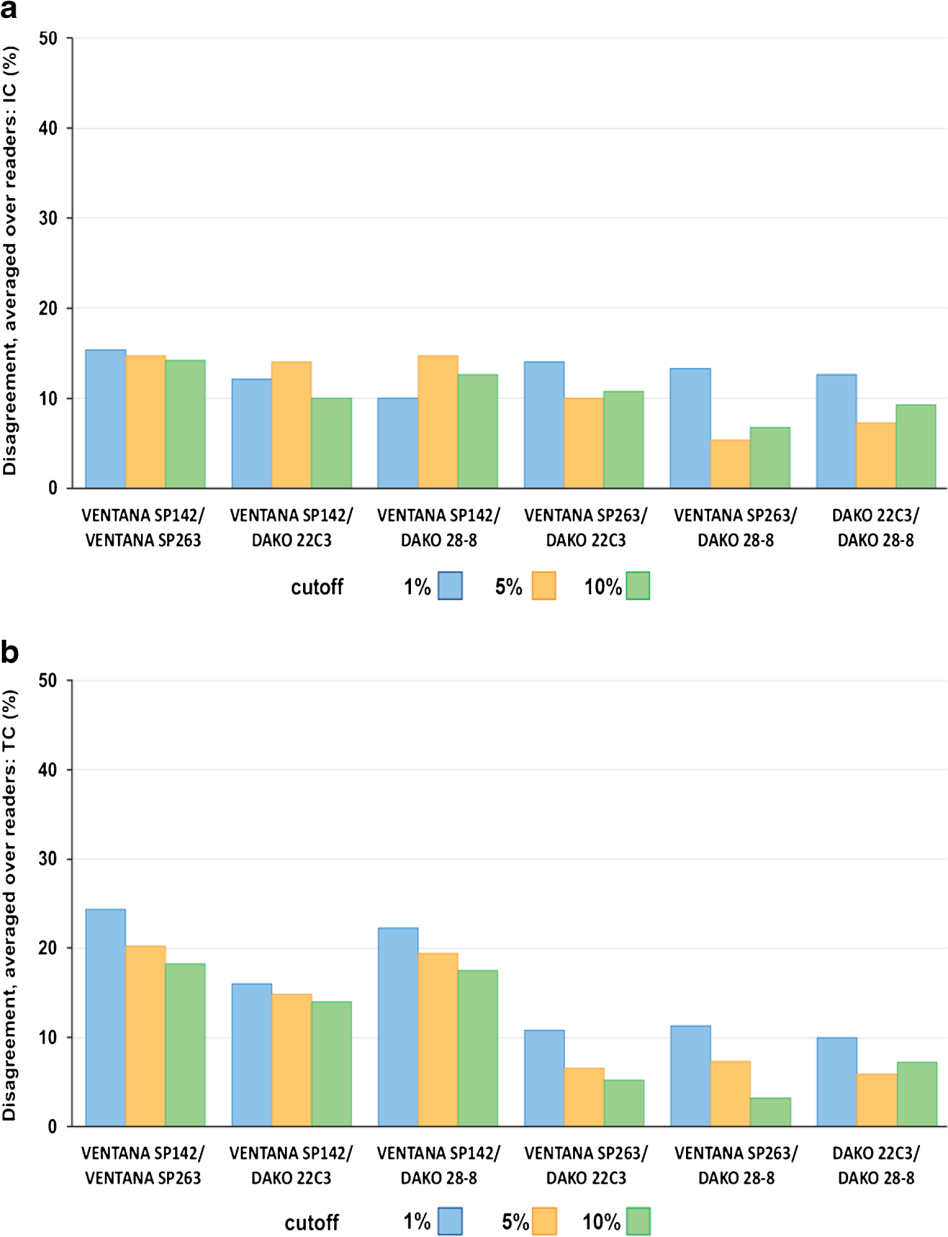
Percentage of disagreement between assays averaged across five readers when results were allocated to retrospective binary cutoffs for PD-L1-stained IC (**a**) or TC (**b**). IC = tumor-infiltrating immune cells; PD-L1 = programmed death-ligand 1; TC = tumor cells

When IC results reported by each reader were allocated to cutoffs of 1%, 5%, or 10%, which have been used previously [6], average agreement between assays was high, with fewer than 15% of cases giving discordant results for any two assays (Fig. 3a). In contrast, when TC results were allocated to the same cutoffs (1%, 5%, and 10%), up to 25% of cases showed discordant results in comparisons involving VENTANA SP142 (Fig. 3b). When VENTANA SP142 was excluded, the average agreement between the other assays was high (> 88%).

For PD-L1-stained IC, inter-assay agreement appeared highest at the lowest cutoff point, with Kappa values ranging from 0.609 to 0.923 for > 1%, from 0.683 to 0.811 for > 5%, and from 0.440 to 0.763 for > 10% (Table S3 in the supplementary material). For inter-reader agreement, Kappa values ranged from 0.533 to 0.801 for > 1%, from 0.551 to 0.732 for > 5%, and from 0.343 to 0.706 for > 10% (Table S4 in the supplementary material). For PD-L1-stained TC, no cutoff appeared to give greater agreement than any other. Inter-assay agreement showed Kappa values ranging from 0.560 to 0.844 (Table S5 in the supplementary material) and inter-reader agreement ranging from 0.572 to 0.769 (Table S6 in the supplementary material).

## Discussion

Cancer immunotherapy, and specifically PD-L1/PD-1 inhibition, is an effective treatment option for difficult-to-treat, locally advanced, or metastatic cancers, including UBC.

Currently there are several different PD-L1 antibodies and assays in use, developed in conjunction with different checkpoint inhibitor studies, and the technical parameters vary. This limits comparison of the different trials with respect to outcomes in the PD-L1 biomarker-positive subgroups and use of the biomarker in clinical practice.

Regarding PD-L1 IC staining, several seminal studies have shown lower concordance between readers or assays [17, 20, 21]. It was suggested that IC PD-L1 scoring is more difficult and thus may require further standardization and training before using IC-based algorithms for patient selection [17, 20–22].

In this study, ahead of reading the slides stained with the different assays, all readers attended classroom training for scoring PD-L1-stained IC (per tumor area) with SP142 in UBC [18]. The consistency of results for IC staining suggests that the percentage of PD-L1-stained IC per tumor area can be evaluated reproducibly by trained readers.

For IC staining we found little variation between assays, with small, non-significant differences and medium to high ICC values for inter-assay agreement. For TC staining, on the other hand, the VENTANA SP142 IHC assay gave consistently lower percentages of PD-L1-stained cells compared with the other three assays, as has been shown previously in non-small-cell lung cancer [17, 20, 21] and UBC [23]. These differences in staining could be explained by the fact that this assay was specifically designed to stain IC and compared with the other three antibodies; some of its binding epitopes are absent in the PD-L1 isoform 2 [24]. Excluding VENTANA SP142, adjusted mean differences for TC were small and non-significant.

Previously, it has been suggested that the four assays yield substantial to high correlations for PD-L1 IC positivity read by trained readers in UBC [23, 25–27]. These studies were based on scores from one, two, or four readers who had read core tissue microarrays. In the study with four readers, the scores were consented before analysis [25]. Interestingly, some studies [26, 27], which were based on core tissue microarrays, have reported possibly lower IC sensitivity for SP142, which may reflect a sampling bias and intra-tumor heterogeneity. To account for tumor heterogeneity and subsequently more subjective interpretations [28, 29], we used whole slides for analysis. Also, to prevent potential difficulties in differentiating TC from IC, an additional pan-cytokeratin stain was included for each case. In addition, we based our assay comparisons on five independent readers, who were blinded for the PD-L1 assay used. For assay comparisons, the results were adjusted for reader effects. Hence, we show for the first time in a clinically relevant setting that the PD-L1 assays SP142, SP263, 22C3, and 28-8 stain similar percentages of PD-L1 IC, with no statistically significant differences between assays.

In regard to overall percentage agreement at defined cutoffs for PD-L1 IC or TC positivity, our study yielded substantial to high agreement values for assay pairs as have been published in larger UBC cohorts [23, 26]. Hence, we assume that the results from our cohort of 30 patients with UBC are representative of larger UBC patient sets. However, the current analysis is exploratory, with a small sample size, and there was no formal testing of equivalence. To formally confirm the analytical similarity of the assays, comparison of computerized evaluations on digitalized slides would be desirable; future studies should address this.

As previously reported [26, 30], we could also see lower inter-reader agreement at lower cutoffs (> 1%) for TC scoring using VENTANA SP142, DAKO 28-8, and 22C3. This might be due to the fact that, in interpretation of PD-L1 staining, even faintly and non-circumferentially stained tumor cells are considered positive, whereas in HER2 testing in breast cancer this type of staining is considered negative. Regarding IC scoring, VENTANA SP142 and DAKO 22C3 also yielded lower inter-reader agreement at lower cutoffs (< 1%). On the other hand, we could detect a decline in inter-reader agreement for IC scoring at higher cutoffs (> 10%) for VENTANA SP263 and DAKO 28-8.

In the current study, no correlation of staining with clinical outcomes could be attempted, as the samples were not taken from patients treated with anti-PD-L1/PD-1 therapy. While the cohort size was sufficient to detect the relatively large differences in TC staining between SP142 and the other assays, it is not known if other, more subtle, differences would become detectable in a larger cohort. But even if they exist, it is unclear whether such minor discrepancies would have any clinical impact. One limitation is that the experienced readers in this study may have recognized subtle characteristic staining features of the different antibodies (such as the more granular staining pattern of VENTANA SP142), so true blinding may not have been possible.

This is the first multicenter comparison of assay performance and inter-observer agreement for PD-L1 testing in UBC using all currently diagnostically relevant assays by readers trained on scoring PD-L1-stained IC on whole slides. The results from 30 patients suggest that, in advanced UBC, the four assays may be considered analytically similar for assessing the percentage of PD-L1-stained IC per tumor area. In addition, three of the assays (VENTANA SP263, DAKO 22C3, and DAKO 28-8) may be considered analytically similar for assessing the percentage of PD-L1-stained TC. Our data facilitate the clinical use of the biomarker PD-L1 as it contributes to the understanding of the technical comparability and need for training in IC PD-L1 testing and scoring in UBC.

## Electronic supplementary material

ESM 1 (PDF 844 kb)

## Data Availability

Data sets generated during this study are available from the corresponding author upon reasonable request. Digital images of all cases stained with H&E, pan-cytokeratin, or any of the four PD-L1 assays are available online at http://www.roche.de/pdl1testing. For further detail on Roche’s Global Policy on the Sharing of Clinical Information and how to request access to related clinical study documents, see here: https://www.roche.com/research_and_development/who_we_are_how_we_work/clinical_trials/our_commitment_to_data_sharing.htm.

## References

[1] Antoni S, Ferlay J, Soerjomataram I, Znaor A, Jemal A, Bray F (2017). Bladder cancer incidence and mortality: a global overview and recent trends. Eur Urol.

[2] Necchi Andrea, Sonpavde Guru, Lo Vullo Salvatore, Giardiello Daniele, Bamias Aristotelis, Crabb Simon J., Harshman Lauren C., Bellmunt Joaquim, De Giorgi Ugo, Sternberg Cora N., Cerbone Linda, Ladoire Sylvain, Wong Yu-Ning, Yu Evan Y., Chowdhury Simon, Niegisch Gunter, Srinivas Sandy, Vaishampayan Ulka N., Pal Sumanta K., Agarwal Neeraj, Alva Ajjai, Baniel Jack, Golshayan Ali-Reza, Morales-Barrera Rafael, Bowles Daniel W., Milowsky Matthew I., Theodore Christine, Berthold Dominik R., Daugaard Gedske, Sridhar Srikala S., Powles Thomas, Rosenberg Jonathan E., Galsky Matthew D., Mariani Luigi (2017). Nomogram-based Prediction of Overall Survival in Patients with Metastatic Urothelial Carcinoma Receiving First-line Platinum-based Chemotherapy: Retrospective International Study of Invasive/Advanced Cancer of the Urothelium (RISC). European Urology.

[3] De Santis M, Bellmunt J, Mead G, Kerst JM, Leahy M, Maroto P, Gil T, Marreaud S, Daugaard G, Skoneczna I, Collette S, Lorent J, de Wit R, Sylvester R (2012). Randomized phase II/III trial assessing gemcitabine/carboplatin and methotrexate/carboplatin/vinblastine in patients with advanced urothelial cancer who are unfit for cisplatin-based chemotherapy: EORTC study 30986. J Clin Oncol.

[4] von der Maase H, Sengelov L, Roberts JT, Ricci S, Dogliotti L, Oliver T, Moore MJ, Zimmermann A, Arning M (2005). Long-term survival results of a randomized trial comparing gemcitabine plus cisplatin, with methotrexate, vinblastine, doxorubicin, plus cisplatin in patients with bladder cancer. J Clin Oncol.

[5] Chen DS, Mellman I (2013). Oncology meets immunology: the cancer-immunity cycle. Immunity.

[6] Balar AV, Galsky MD, Rosenberg JE, Powles T, Petrylak DP, Bellmunt J, Loriot Y, Necchi A, Hoffman-Censits J, Perez-Gracia JL, Dawson NA, van der Heijden MS, Dreicer R, Srinivas S, Retz MM, Joseph RW, Drakaki A, Vaishampayan UN, Sridhar SS, Quinn DI, Duran I, Shaffer DR, Eigl BJ, Grivas PD, Yu EY, Li S, Kadel EE, Boyd Z, Bourgon R, Hegde PS, Mariathasan S, Thastrom A, Abidoye OO, Fine GD, Bajorin DF, IMvigor210 Study Group (2017). Atezolizumab as first-line treatment in cisplatin-ineligible patients with locally advanced and metastatic urothelial carcinoma: a single-arm, multicentre, phase 2 trial. Lancet.

[7] Bellmunt J, de Wit R, Vaughn DJ, Fradet Y, Lee JL, Fong L, Vogelzang NJ, Climent MA, Petrylak DP, Choueiri TK, Necchi A, Gerritsen W, Gurney H, Quinn DI, Culine S, Sternberg CN, Mai Y, Poehlein CH, Perini RF, Bajorin DF, KEYNOTE-045 Investigators (2017). Pembrolizumab as second-line therapy for advanced urothelial carcinoma. N Engl J Med.

[8] Rosenberg JE, Hoffman-Censits J, Powles T, van der Heijden MS, Balar AV, Necchi A, Dawson N, O’Donnell PH, Balmanoukian A, Loriot Y, Srinivas S, Retz MM, Grivas P, Joseph RW, Galsky MD, Fleming MT, Petrylak DP, Perez-Gracia JL, Burris HA, Castellano D, Canil C, Bellmunt J, Bajorin D, Nickles D, Bourgon R, Frampton GM, Cui N, Mariathasan S, Abidoye O, Fine GD, Dreicer R (2016). Atezolizumab in patients with locally advanced and metastatic urothelial carcinoma who have progressed following treatment with platinum-based chemotherapy: a single-arm, multicentre, phase 2 trial. Lancet.

[9] Necchi A, Joseph RW, Loriot Y, Hoffman-Censits J, Perez-Gracia JL, Petrylak DP, Derleth CL, Tayama D, Zhu Q, Ding B, Kaiser C, Rosenberg JE (2017). Atezolizumab in platinum-treated locally advanced or metastatic urothelial carcinoma: post-progression outcomes from the phase II IMvigor210 study. Ann Oncol.

[10] Powles T, Eder JP, Fine GD, Braiteh FS, Loriot Y, Cruz C, Bellmunt J, Burris HA, Petrylak DP, Teng SL, Shen X, Boyd Z, Hegde PS, Chen DS, Vogelzang NJ (2014). MPDL3280A (anti-PD-L1) treatment leads to clinical activity in metastatic bladder cancer. Nature.

[11] Balar AV, Castellano D, O’Donnell PH, Grivas P, Vuky J, Powles T, Plimack ER, Hahn NM, de Wit R, Pang L, Savage MJ, Perini RF, Keefe SM, Bajorin D, Bellmunt J (2017). First-line pembrolizumab in cisplatin-ineligible patients with locally advanced and unresectable or metastatic urothelial cancer (KEYNOTE-052): a multicentre, single-arm, phase 2 study. Lancet Oncol.

[12] Powles T, Durán I, van der Heijden MS, Loriot Y, Vogelzang NJ, De Giorgi U, Oudard S, Retz MM, Castellano D, Bamias A (2018). Atezolizumab versus chemotherapy in patients with platinum-treated locally advanced or metastatic urothelial carcinoma (IMvigor211): a multicentre, open-label, phase 3 randomised controlled trial. Lancet.

[13] Genentech, Inc. (2018) TECENTRIQ® (atezolizumab). Prescribing information. https://www.accessdata.fda.gov/drugsatfda_docs/label/2018/761034s012lbl.pdf. Accessed 18 Feb 2019

[14] US Food and Drug Administration (FDA) (2019) KEYTRUDA® (pembrolizumab). Prescribing information. https://www.accessdata.fda.gov/drugsatfda_docs/label/2019/125514s040lbl.pdf. Accessed 18 Feb 2019

[15] European Medicines Agency (EMA) (2018) EMA restricts use of Keytruda and Tecentriq in bladder cancer [press release]. 1 June 2018. https://www.ema.europa.eu/en/news/ema-restricts-use-keytruda-tecentriq-bladder-cancer. Accessed 18 Feb 2019

[16] US Food and Drug Administration (FDA) (2018) FDA alerts health care professionals and oncology clinical investigators about an efficacy issue identified in clinical trials for some patients taking Keytruda (pembrolizumab) or Tecentriq (atezolizumab) as monotherapy to treat urothelial cancer with low expression of PD-L1 [press release]. 16 August 2018. https://www.fda.gov/Drugs/DrugSafety/ucm608075.htm. Accessed 18 Feb 2019

[17] Hirsch FR, McElhinny A, Stanforth D, Ranger-Moore J, Jansson M, Kulangara K, Richardson W, Towne P, Hanks D, Vennapusa B, Mistry A, Kalamegham R, Averbuch S, Novotny J, Rubin E, Emancipator K, McCaffery I, Williams JA, Walker J, Longshore J, Tsao MS, Kerr KM (2017). PD-L1 immunohistochemistry assays for lung cancer: results from phase 1 of the Blueprint PD-L1 IHC assay comparison project. J Thorac Oncol.

[18] Vennapusa B, Baker B, Kowanetz M, Boone J, Menzl I, Bruey JM, Fine G, Mariathasan S, McCaffery I, Mocci S, Rost S, Smith D, Dennis E, Tang SY, Damadzadeh B, Walker E, Hegde PS, Williams JA, Koeppen H, Boyd Z (2019). Development of a PD-L1 complementary diagnostic immunohistochemistry assay (SP142) for atezolizumab. Appl Immunohistochem Mol Morphol.

[19] Dennis E, Vennapusa B, ElGabry E, Walker E, Smith D, Cardona J, Kockx M (2017). Robust and reproducible pathologist training for PD-L1 assessing tumour cells (TC) and immune cells (IC) utilising novel digital training platform. Virchows Arch.

[20] Rimm DL, Han G, Taube JM, Eunhee SY, Bridge JA, Flieder DB, Homer R, West WW, Wu H, Roden AC (2017). A prospective, multi-institutional, pathologist-based assessment of 4 immunohistochemistry assays for PD-L1 expression in non-small cell lung cancer. JAMA Oncol.

[21] Scheel AH, Dietel M, Heukamp LC, Jöhrens K, Kirchner T, Reu S, Rüschoff J, Schildhaus HU, Schirmacher P, Tiemann M (2016). Harmonized PD-L1 immunohistochemistry for pulmonary squamous-cell and adenocarcinomas. Mod Pathol.

[22] Büttner R, Gosney JR, Skov BG, Adam J, Motoi N, Bloom KJ, Dietel M, Longshore JW, López-Ríos F, Penault-Llorca F (2017). Programmed death-ligand 1 immunohistochemistry testing: a review of analytical assays and clinical implementation in non-small-cell lung cancer. J Clin Oncol.

[23] Hodgson A, Slodkowska E, Jungbluth A, Liu SK, Vesprini D, Enepekides D, Higgins K, Katabi N, Xu B, Downes MR (2018). PD-L1 immunohistochemistry assay concordance in urothelial carcinoma of the bladder and hypopharyngeal squamous cell carcinoma. Am J Surg Pathol.

[24] Schats Kelly, Van Vre Emily A., Schrijvers Dorien, De Meester Ingrid, Kockx Mark (2017). Epitope mapping of PD-L1 primary antibodies (28-8, SP142, SP263, E1L3N). Journal of Clinical Oncology.

[25] Zavalishina L, Tsimafeyeu I, Povilaitite P, Raskin G, Andreeva Y, Petrov A, Kharitonova E, Rumyantsev A, Pugach I, Frank G (2018). RUSSCO-RSP comparative study of immunohistochemistry diagnostic assays for PD-L1 expression in urothelial bladder cancer. Virchows Arch.

[26] Eckstein M, Erben P, Kriegmair MC, Worst TS, Weiß CA, Wirtz RM, Wach S, Stoehr R, Sikic D, Geppert CI, Weyerer V, Bertz S, Breyer J, Otto W, Keck B, Burger M, Taubert H, Weichert W, Wullich B, Bolenz C, Hartmann A, Erlmeier F (2019). Performance of the Food and Drug Administration/EMA-approved programmed cell death ligand-1 assays in urothelial carcinoma with emphasis on therapy stratification for first-line use of atezolizumab and pembrolizumab. Eur J Cancer.

[27] Tretiakova M, Fulton R, Kocherginsky M, Long T, Ussakli C, Antic T, Gown A (2018). Concordance study of PD-L1 expression in primary and metastatic bladder carcinomas: comparison of four commonly used antibodies and RNA expression. Mod Pathol.

[28] Casadevall D, Clavé S, Taus Á, Hardy-Werbin M, Rocha P, Lorenzo M, Menéndez S, Salido M, Albanell J, Pijuan L (2017). Heterogeneity of tumor and immune cell PD-L1 expression and lymphocyte counts in surgical NSCLC samples. Clin Lung Cancer.

[29] Scott ML, Scorer P, Lawson N, Ratcliffe MJ, Barker C, Rebelatto M, Walker J (2017). Assessment of heterogeneity of PD-L1 expression in NSCLC, HNSCC, and UC with Ventana SP263 assay. J Clin Oncol.

[30] Ratcliffe MJ, Sharpe A, Midha A, Barker C, Scott M, Scorer P, Al-Masri H, Rebelatto MC, Walker J (2017). Agreement between programmed cell death ligand-1 diagnostic assays across multiple protein expression cutoffs in non-small cell lung cancer. Clin Cancer Res.

