# Supplementary material for: Multicentric analytical comparability study of programmed death-ligand 1 expression on tumor-infiltrating immune cells and tumor cells in urothelial bladder cancer using four clinically developed immunohistochemistry assays
Source: Virchows Arch. 2019 Jul 2;475(5):599–608. doi: 10.1007/s00428-019-02610-z (PMC6861354; doi:10.1007/s00428-019-02610-z)
Supplement: Supplementary file 1 — (PDF 844 kb) [file 428_2019_2610_MOESM1_ESM.pdf]

Multicentric analytical comparability study of programmed death-ligand 1 expression on tumor-infiltrating immune cells and tumor cells in urothelial bladder cancer using four clinically developed immunohistochemistry assays

K Schwamborn *et al*.

Electronic supplementary material

Fig. S1. Example staining for (**a**) PD-L1-stained IC (5%) and (**b**) PD-L1-negative IC, with each assay. H&E = hematoxylin and eosin; IC = tumor-infiltrating immune cells; PD-L1 = programmed death-ligand 1

For readers of this publication, digital images of all cases stained with H&E, pan-cytokeratin or either of the four PD-L1 assays are available online at http://www.roche.de/pdl1testing

a

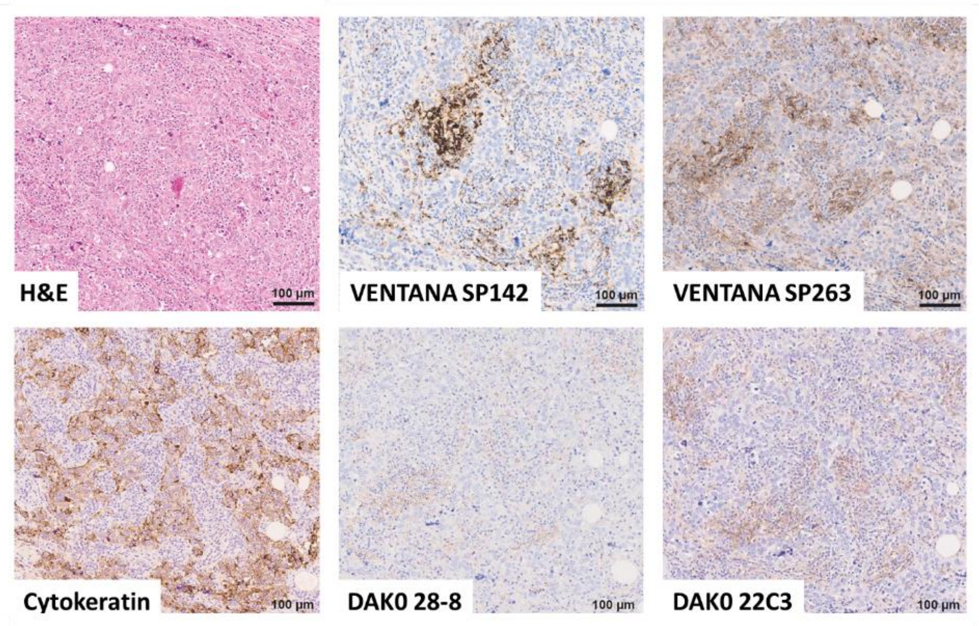

b

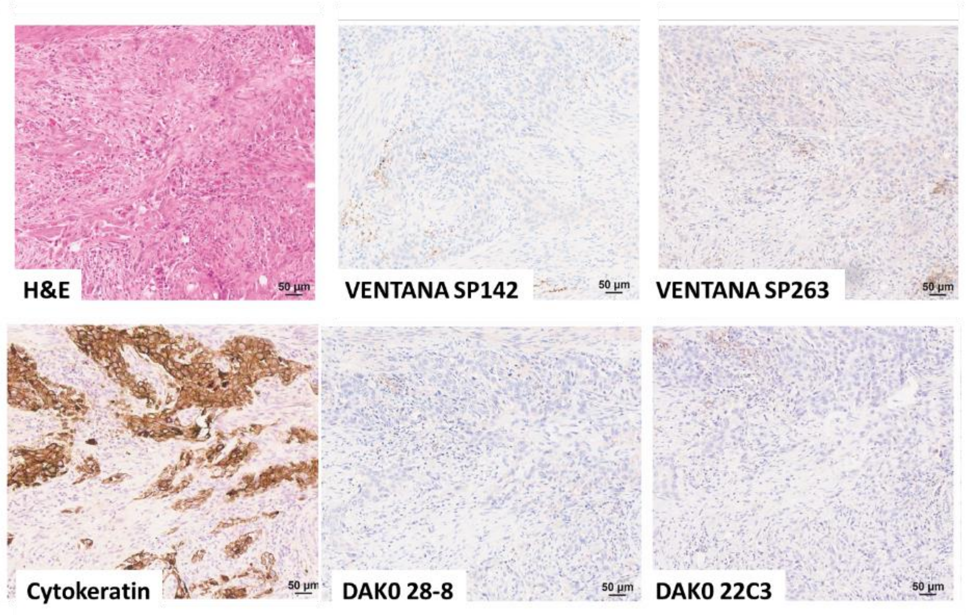

Fig. S2. Percentage of PD-L1-stained IC for each assay and each reader. IC = tumor-infiltrating immune cells; PD-L1 = programmed death-ligand 1; R = reader

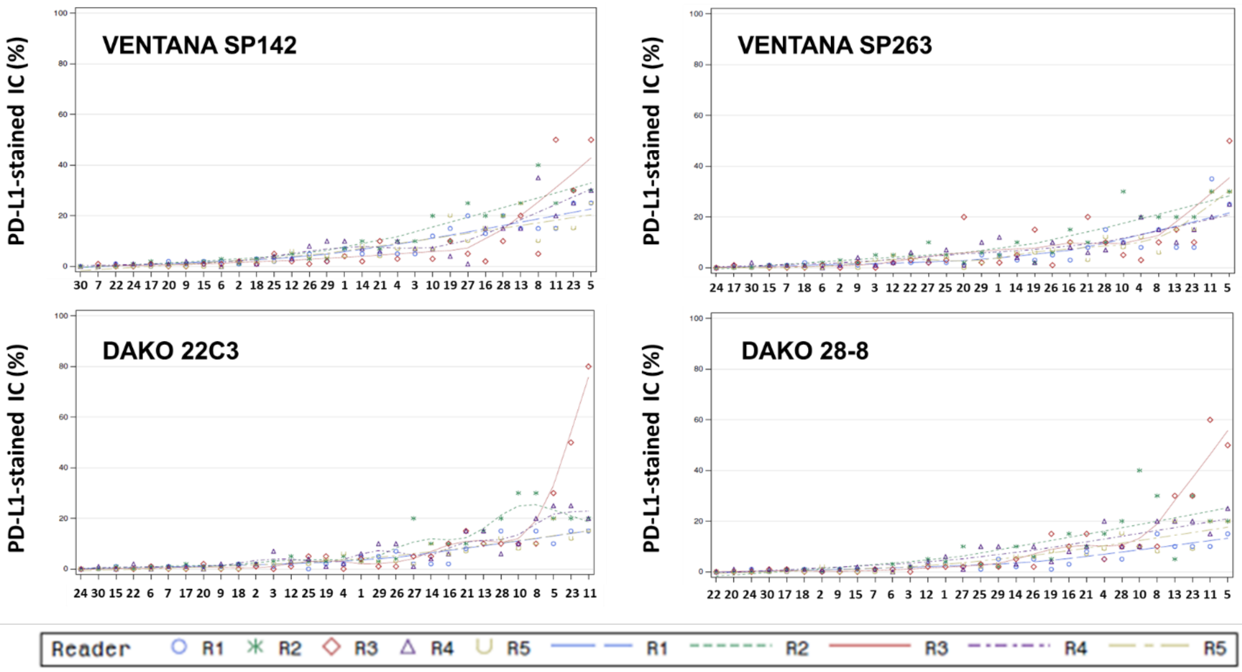

Fig. S3. Percentage of PD-L1-stained TC for each assay and each reader. PD-L1 = programmed death-ligand 1; TC = tumor cells

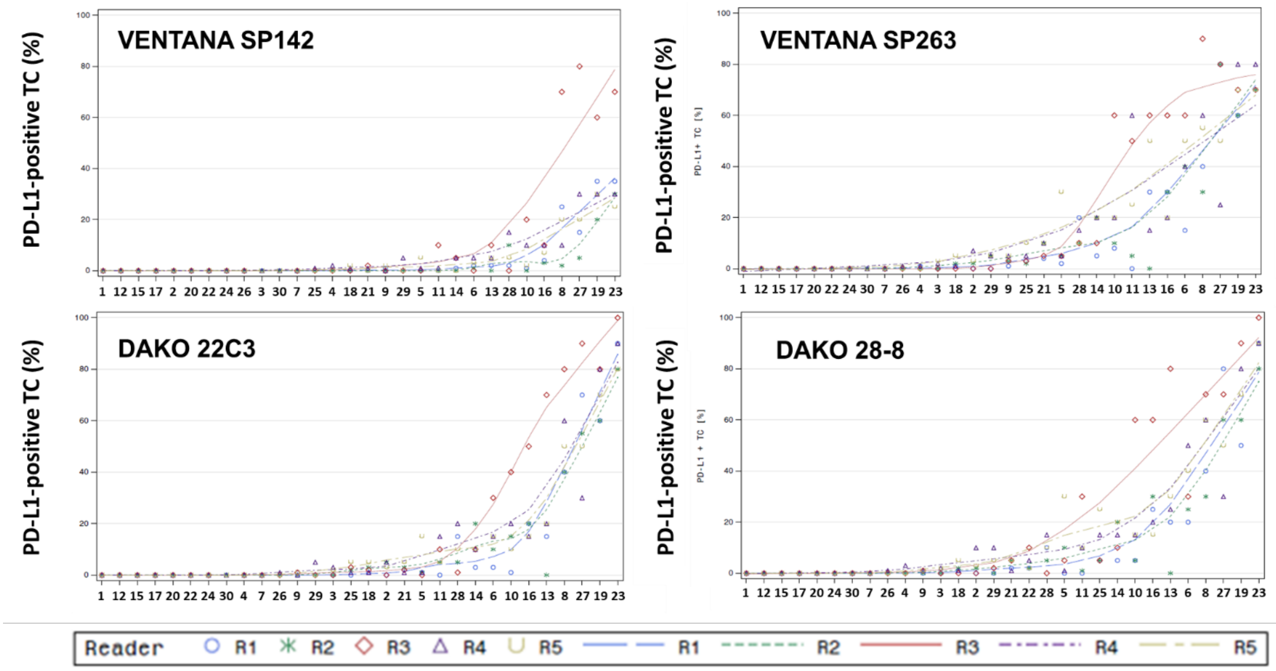

Table S1. Characteristics and clinical features of cases

**Patients, *n* (%)**	**<1%**<br>***n* = 10**	**1–5%**<br>***n* = 10**	**>5%**<br>***n* = 10**	**All**<br>***N* = 30**
**Biopsy type**				
Cystectomy	8 (80)	7 (70)	7 (7)	22 (73)
Transurethral resection	2 (20)	3 (30)	3 (30)	8 (27)
**TNM classification**	3 (30)	4 (40)	3 (30)	10 (33)
pT2	7 (70)	6 (60)	7 (70)	20 (67)
pT3+				
**BCG pre-treatment**				
Yes	1 (10)	1 (10)	0 (0)	2 (7)
No	9 (90)	8 (80)	4 (40)	21 (70)
Information missing	0 (0)	1 (10)	6 (60)	7 (23)

TNM = tumour, node, and metastasis; BCG = Bacillus Calmette–Guérin

Table S2. Differences in adjusted means between assays for PD-L1-stained IC and TC

Assay pair	Difference in adjusted means (95% CI)	*P* value
**PD-L1-positive IC**		
VENTANA SP142 – VENTANA SP263	1.1 (–0.6 to 2.8)	0.3500
VENTANA SP142 – DAKO 22C3	1.6 (–0.1 to 3.3)	0.0646
VENTANA SP142 – DAKO 28-8	1.3 (–0.4 to 3.0)	0.2029
VENTANA SP263 – DAKO 22C3	0.5 (–1.2 to 2.2)	0.8442
VENTANA SP263 – DAKO 28-8	0.2 (–1.5 to 1.9)	0.9900
DAKO 22C3 – DAKO 28-8	–0.3 (–2.0 to 1.4)	0.9548
**PD-L1-positive TC**		
Assay pair	Difference in adjusted means (95% CI)	*P* value
VENTANA SP142 – VENTANA SP263	–10.5 (–13.7 to –7.3)	<0.0001
VENTANA SP142 – DAKO 22C3	–7.8 (–11.0 to –4.6)	<0.0001
VENTANA SP142 – DAKO 28-8	–9.7 (–12.9 to –6.5)	<0.0001
VENTANA SP263 – DAKO 22C3	2.7 (–0.5 to 5.9)	0.1325
VENTANA SP263 – DAKO 28-8	0.8 (–2.4 to 3.9)	0.9251
DAKO 22C3 – DAKO 28-8	–1.9 (–5.1 to 1.3)	0.4065

CI = confidence interval; IC = tumor-infiltrating immune cells; PD-L1 = programmed death-ligand 1; TC = tumor cells.

Table S3. Inter-assay agreement Kappa values for each reader at different cutoffs for IC

PD-L1-positive IC			
Reader	Cutoff points (%)	*n*	Kappa (95% CI)
R1	>1	30	0.642 (0.496–0.788)
	>5	30	0.683 (0.536–0.829)
	>10	30	0.440 (0.294–0.586)
R2	>1	30	0.609 (0.462–0.755)
	>5	30	0.751 (0.605–0.897)
	>10	30	0.763 (0.617–0.909)
R3	>1	27	0.672 (0.518–0.826)
	>5	27	0.763 (0.609–0.917)
	>10	27	0.609 (0.455–0.763)
R4	>1	30	0.633 (0.487–0.779)
	>5	30	0.811 (0.665–0.957)
	>10	30	0.680 (0.534–0.826)
R5	>1	30	0.923 (0.333–1.000)
	>5	30	0.771 (0.625–0.918)
	>10	30	0.711 (0.565–0.857)

CI = confidence interval; IC = tumor-infiltrating immune cells; PD-L1 = programmed death-ligand 1; R = reader

Table S4. Inter-reader agreement Kappa values for each assay at different cutoffs for IC

PD-L1-positive IC			
Assay	Cutoff points (%)	*n*	Kappa (95% CI)
VENTANA SP142	>1	28	0.609 (0.492–0.726)
	>5	28	0.575 (0.458–0.692)
	>10	28	0.706 (0.589–0.823)
VENTANA SP263	>1	28	0.685 (0.568–0.802)
	>5	28	0.551 (0.434–0.668)
	>10	28	0.343 (0.225–0.460)
DAKO 22C3	>1	29	0.533 (0.418–0.648)
	>5	29	0.732 (0.617–0.847)
	>10	29	0.578 (0.463–0.693)
DAKO 28-8	>1	30	0.801 (0.687–0.914)
	>5	30	0.630 (0.516–0.743)
	>10	30	0.418 (0.305–0.532)

CI = confidence interval; IC = tumor-infiltrating immune cells; PD-L1 = rogrammed death-ligand 1

Table S5. Inter-assay agreement Kappa values for each reader at different cutoffs for TC

PD-L1-positive TC			
Reader	Cutoff points (%)	*n*	Kappa (95% CI)
R1	>1	30	0.761 (0.615–0.907)
	>5	30	0.752 (0.605–0.898)
	>10	30	0.771 (0.625–0.917)
R2	>1	30	0.570 (0.424–0.716)
	>5	30	0.560 (0.414–0.707)
	>10	30	0.659 (0.513–0.805)
R3	>1	28	0.702 (0.550–0.853)
	>5	28	0.844 (0.693–0.996)
	>10	28	0.807 (0.656–0.958)
R4	>1	30	0.678 (0.532–0.824)
	>5	30	0.704 (0.558–0.850)
	>10	30	0.656 (0.510–0.802)
R5	>1	30	0.673 (0.527–0.819)
	>5	30	0.650 (0.504–0.796)
	>10	30	0.644 (0.498–0.790)

CI = confidence interval; PD-L1 = programmed death-ligand 1; R = reader; TC = tumor cells

Table S6. Inter-reader agreement Kappa values for each assay at different cutoffs for TC

**PD-L1-positive TC**			
**Assay**	**Cutoff points (%)**	***n***	**Kappa (95% CI)**
VENTANA SP142	>1	28	0.572 (0.455–0.689)
	>5	28	0.610 (0.493–0.727)
	>10	28	0.726 (0.609–0.843)
VENTANA SP263	>1	29	0.747 (0.632–0.862)
	>5	29	0.769 (0.654–0.884)
	>10	29	0.745 (0.630–0.860)
DAKO 22C3	>1	29	0.640 (0.525–0.755)
	>5	29	0.748 (0.633–0.863)
	>10	29	0.707 (0.591–0.822)
DAKO 28-8	>1	30	0.652 (0.539–0.766)
	>5	30	0.638 (0.525–0.751)
	>10	30	0.730 (0.617–0.843)

CI = confidence interval; PD-L1 = programmed death-ligand 1; TC = tumor cells
